# A New Approach for Calculating Texture Coefficients of Different Rocks With Image Segmentation and Image Processing Techniques

**DOI:** 10.1002/jemt.24879

**Published:** 2025-05-26

**Authors:** Emre Karakaya, Bilgehan Kekeç, Niyazi Bilim, Fatih V. Adigözel

**Affiliations:** ^1^ Faculty of Engineering and Natural Sciences Konya Technical University Konya Türkiye

**Keywords:** image processing, image segmentation, rock texture, texture coefficient, thin‐section image

## Abstract

The texture coefficient (TC) is a critical parameter used to analyze the microstructural characteristics of rocks and predict their mechanical behavior. In recent years, various computational programs and software have been employed to estimate the TC values of rocks. However, existing methods remain insufficient and time‐consuming for accurately determining rock TCs. In this study, thin‐section images of 20 different igneous, metamorphic, and sedimentary rocks were acquired and segmented to calculate TC values using a novel approach. The computation process was implemented using Python‐based software that integrates segmentation and image processing techniques to determine TC values. The thin‐section images were segmented utilizing a deep learning‐based image processing technique, and a Python‐based algorithm was developed for TC calculations. The proposed method offers a unique approach to TC estimation in rocks, achieving a high segmentation accuracy (IoU = 0.97). Furthermore, with this method, the TC value of any given rock can be computed in approximately 1 min.

## Graphical abstract

This study calculated the texture coefficient (TC) values of 20 different rock textures with magmatic, metamorphic, and sedimentary origins, which underwent image segmentation using a novel approach.

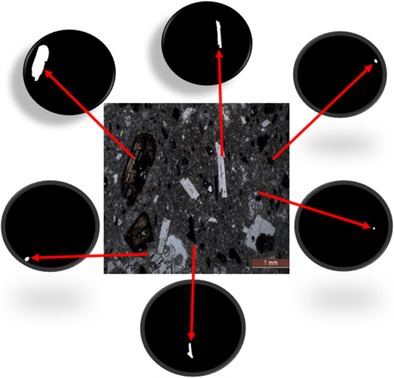

**Summary**

- The texture coefficient (TC) is a critical parameter for the determination of rock properties.
- In recent years, various computer programs and software have been employed to estimate the TC value of rocks.
- However, the existing programs and software used to determine the TC of rocks are inadequate and time‐consuming.
- This study calculated the TC values of 20 different rock textures with magmatic, metamorphic, and sedimentary origin, which underwent image segmentation using a novel approach.
- The calculation process was implemented using Python software, employing segmentation and image processing techniques to find the TC values of rocks.
- Thin‐section images of rocks were segmented using a deep learning‐based image processing technique, and software was developed in Python to perform TC calculations for rocks.
- The proposed method for TC calculations in rocks is highly unique, and the segmentation accuracy rate of this method (IoU = 0.97) is remarkably high.
- Moreover, calculating the TC value of any rock with this proposed method takes approximately 1 min.

## 1 Introduction

In contemporary times, advancements in image processing techniques are increasingly finding applications in various fields, including the earth sciences. Many researchers employ image processing techniques to address geological challenges or facilitate engineering and practical applications in the earth sciences. Examples of such applications include the investigation of the solid‐porosity relationship and determination of the distribution ratios of feldspars in rocks (Passas et al. 1996), analysis of the particle distribution and geometry of crushed aggregate materials (Wang 1999), determination of the petrographic characteristics of marbles (Lumbreras and Serrat 1996), exploration of surface roughness and aperture in rock fractures, especially using laser light sources (Lanaro 2000), and various studies such as a numerical image analysis‐based surface gloss measurement system.

Moreover, some studies have applied image processing methods in the natural stone sector. These studies encompass color identification of natural stones (Gökay and Gundogdu 2008), with a significant portion focusing on the classification and pattern recognition of processed natural stone products (Deviren et al. 2000; Carrino et al. 2002). Additionally, image analysis methods have been utilized to compare the glossiness of certain types of marbles (Erdogan 2000).

Numerous studies have been conducted, particularly in rock classification and recognition applications, based on image processing and artificial neural networks. Some of these studies include:

It employed machine learning technology and computer vision techniques to classify, detect, and segment rocks in the Atacama Desert (Thompson et al. 2005). A texture recognition method is proposed based on artificial neural networks trained with limestone carbonate images from southern Italy (Marmo et al. 2005). Some research and applications were performed on the rock type classification model by training on gray images of rock slices using image processing methods (Cheng et al. 2013). A classification method based on a 6D feature space was utilized in mathematical morphology and image processing techniques for rock classification (Młynarczuk et al. 2013). To identify matrices, pores, and rock particles, the grayscale rock images using 3D X‐ray tomography were evaluated for the accuracy and performance of machine learning techniques (Chauhan et al. 2016). In another study, a machine learning model was developed for automatically recognizing and classifying rocks with 86.3% accuracy using the natural image of rocks (Dunlop 2006). The proposed method for automatically classifying rock particles is based on a trained artificial neural network with thin‐section images of rocks, achieving a 98% identification accuracy (Cheng and Guo 2017). In another study, the geological strength index was measured using image processing and an artificial neural network based on images of rock joint surfaces (Hong et al. 2017). An unsupervised feature learning method was proposed to define rock properties using texture images (Lepistö et al. 2005). A model was trained and tested for rock identification models using the Inception‐v3 convolutional neural network model with images of granite, breccia, and phyllite through transfer learning (Zhang et al. 2018). In order to evaluate X‐ray fluorescence spectrum data and imaging features, we analyzed rock microscope images using X‐ray fluorescence technology and machine learning algorithms (Rahman et al. 2016). A model was developed for mineral recognition of rocks using artificial neural networks. The proposed model used microscopic rock images (Baykan and Yılmaz 2010). A pattern recognition algorithm was proposed for the semi‐automatic identification of pore types in thin‐section images (Ghiasi‐Freez et al. 2012). These studies demonstrate the diverse applications of image processing and artificial neural networks in geology and rock science.

Depending on laboratory tests and examinations, a texture coefficient (TC) model was proposed based on rocks' penetrability (Howarth and Rowlands 1987). The idea behind the development of this model is based on the concept that the textural structures of rocks create resistance against the formation of cracks under load.

Many researchers have used this equation to determine rocks' TC values. These researches have demonstrated strong correlations between the TC values of rocks and physico‐mechanical properties (rock fragility, uniaxial compressive strength, Brazilian strength, Schmidt hardness, specific energy, and drilling rate). This scientific research shows that the TC value is an important parameter that can be used to predict the strength and penetrability of rocks (Azzoni et al. 1996; Ersoy and Waller 1995; Esmailzadeh et al. 2017; Çomakli and Atici 2017; Çomaklı and Çayırlı 2017; Comakli and Cayirli 2019; Oztürk et al. 2004; Oztürk and Nasuf 2011; Ozturk et al. 2014; Tiryaki and Dikmen 2006; Tumac et al. 2018).

Many researchers have employed various computer programs and software languages (such as Image Pro Plus, JMicro Vision, SigmaScan Pro 5, MATLAB, and Fuzzy Logic) to predict the TC values of rocks (Atici and Comakli 2019; Ozturk and Nasuf 2013; Diamantis et al. 2021; Feng et al. 2021). However, current programs and software used to find the TC of rocks are insufficient and time‐consuming. Table 1 presents various computer programs utilized for TC calculation in rocks, along with their respective image segmentation times, final TC computation durations, total processing times, and corresponding descriptions. The time intervals provided in Table 1 (segmentation time, final TC calculation time, and total time) are based on the assumption that the TC computation is performed practically using rock texture images.

**TABLE 1:** Computer programs used for TC calculation, processing times, and program descriptions.

Software	Segmentation time (minutes)	Final TC calculation time (minutes)	Total time (minutes)	Usage difficulty	Description
Image Pro Plus	5–10	2–3	7–13	Moderate	User‐friendly interface for basic segmentation and texture analyzis; advanced analyzes require additional knowledge.
JMicro Vision	5–10	2–5	7–15	Moderate‐difficult	Suited for microscopic image analyzis; advanced tasks take time and require detailed knowledge.
SigmaScan Pro 5	3–10	2–4	5–14	Moderate	Good for image analyzis, but specific settings must be adjusted for segmentation tasks.
AutoCAD	5–10	4–6	9–16	Difficult	Primarily designed for CAD applications, but can be used for manual segmentation; requires expertise in handling vector‐based images.
MATLAB	2–5	1–2	3–6	Difficult	Highly flexible but requires programming knowledge to write custom scripts for segmentation and texture analyzis.

Upon analyzing Table 1, the difficulty of the programs used for calculating TC, as well as the time spent on image segmentation and TC computation, becomes evident. Furthermore, this time reflects the duration assumed for the practical computation of TC. In addition, despite numerous studies in the literature that have explored relationships between TC values of rocks and various physicomechanical and drillability properties (Azzoni et al. 1996; Ersoy and Waller 1995; Esmailzadeh et al. 2017; Çomakli and Atici 2017; Çomaklı and Çayırlı 2017; Comakli and Cayirli 2019; Oztürk et al. 2004; Oztürk and Nasuf 2011; Ozturk et al. 2014; Tiryaki and Dikmen 2006; Tumac et al. 2018; Khajevand 2021; Askaripour et al. 2022; Fereidooni 2022), no software has been proposed that provides a practical, fast, and straightforward calculation of TC. Therefore, the necessity of calculating the TC parameter, which provides insights into rock strength and drillability properties, in a practical, automatic, less time‐consuming, and easy manner, holds significant scientific importance.

In this study, a total of 20 rock samples were collected from various quarries located in different regions of Anatolia. These samples comprised seven igneous, five sedimentary, five metamorphic, and three metallic rock types. Thin sections were prepared from each sample and mounted on glass slides. Subsequently, three distinct images of each sample were captured using a polarized microscope equipped with a digital camera. The acquired microscopic images were then segmented using a deep learning‐based image processing technique. To facilitate the computation of TC values for the rock samples, a Python‐based software was developed.

## 2 Materials and Methods

### 2.1 Determination of Texture Coefficient

The TC equation proposed by Howarth and Rowlands (1987) has been computed using image processing techniques. Nowadays, this equation is commonly used to determine TC values. However, the calculation of TC values is complex compared to conventional methods. Consequently, providing a straightforward elucidation of the parameters involved in the TC equation is of great significance. Equation (1) presents the TC formula with detailed explanations of its parameters.

(1) $$\begin{array}{c} \mathrm{TC}=\mathrm{AW}\times \;\left[\left(\frac{N_{0}}{N_{0}+N_{1}}\times \frac{1}{{\mathrm{FF}}_{0}}\right)+\left(\frac{N_{1}}{N_{0}+N_{1}}\times {\mathrm{AR}}_{1}\times {\mathrm{AF}}_{1}\right)\right] \end{array}$$

TC = texture coefficient, AW = grain packing weight, *N*_0_ = number of grains whose aspect ratio is below a pre‐set discrimination level, *N*_1_ = number of grains whose aspect ratio is above a pre‐set discrimination level, FF_0_ = arithmetic means of discriminated form‐factors, AR_1_ = arithmetic means of discriminated aspect ratios, AF_1_ = angle factor, quantifying grain orientation.

**FIGURE 1:**
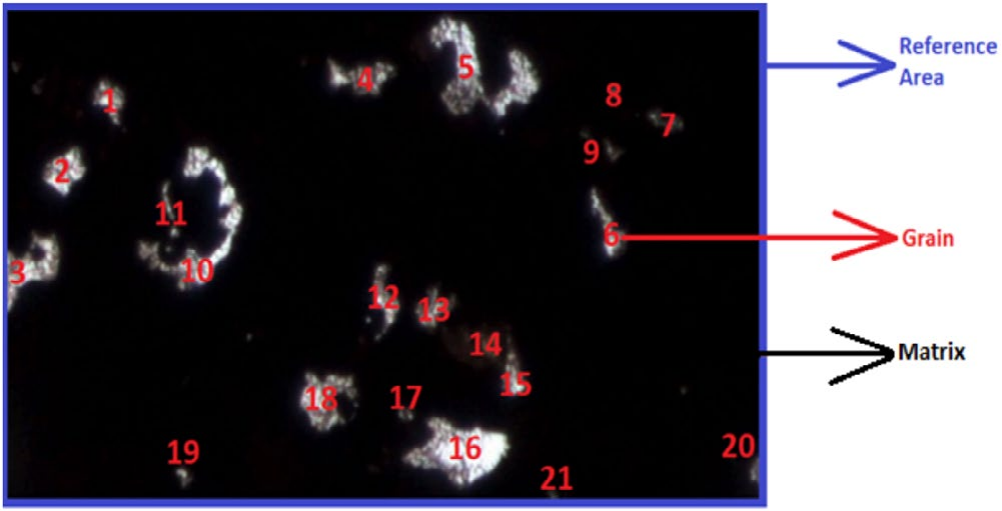
Mineral particles (grain), non‐mineral areas (matrix), and reference areas in microscopic rock image.

AW is obtained by dividing the total area of particles in the image by the area of the entire image. In Figure 1, a microscopic image of a rock texture is presented, illustrating reference areas, the matrix (non‐textured area), and reference area; the matrix and mineral particles have been shown. As seen, there are 21 mineral particles in the image, and the reference area encompasses the entire image. The matrix is cementation among the particles, and it is not considered in the calculation.

To calculate the values of *N*_0_ and *N*_1_, it is necessary first to calculate the aspect ratio of each mineral particle. By numerically determining the aspect ratio (the ratio of width to height) for each mineral particle in the image, the count of particles with an aspect ratio greater than 2 is denoted as *N*_1_, while the count of particles with an aspect ratio less than 2 is denoted as *N*_0_.

**FIGURE 2:**
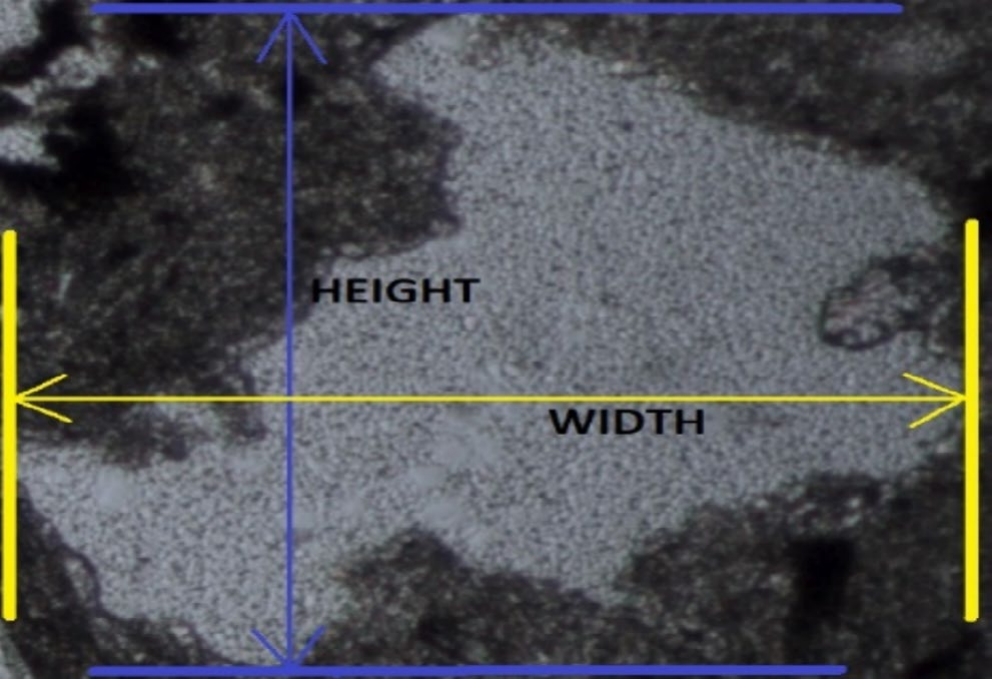
The representation of width and height in any grain.

The value AR_1_ is calculated after analyzing all mineral particles placed in the image. It is calculated by summing the numerical values of particles with an aspect ratio greater than 2 and dividing this sum by *N*_1_ (the count of texture particles with an aspect ratio greater than 2 in the image). Figure 2 illustrates the aspect ratio features of any texture particle in an image of a rock, and the aspect ratio calculation process is applied to all particles.

**FIGURE 3:**
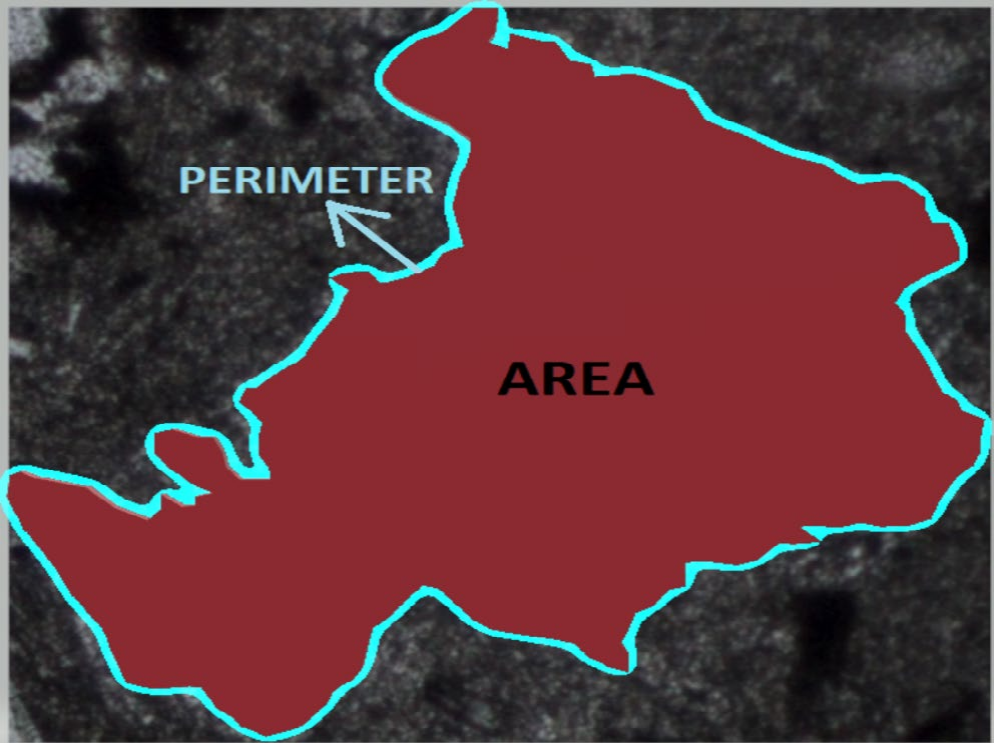
Representation of the area and perimeter in any grain, found in an image of a rock.

The value of FF_0_ is obtained by calculating the form factors of mineral particles with width‐height values less than 2 using the formula in Equation (2) and taking the average of these values. In Figure 3, the area and perimeter of any texture particle in an image of a rock are illustrated. The FF_0_ value is obtained by calculating the area and perimeter of each texture particle in the image.

(2) $${\mathrm{FF}}_{0}=\left(4\times \pi \;\frac{\text{AREA}}{{\text{PERIMETER}}^{2}}\;\right)$$

**FIGURE 4:**
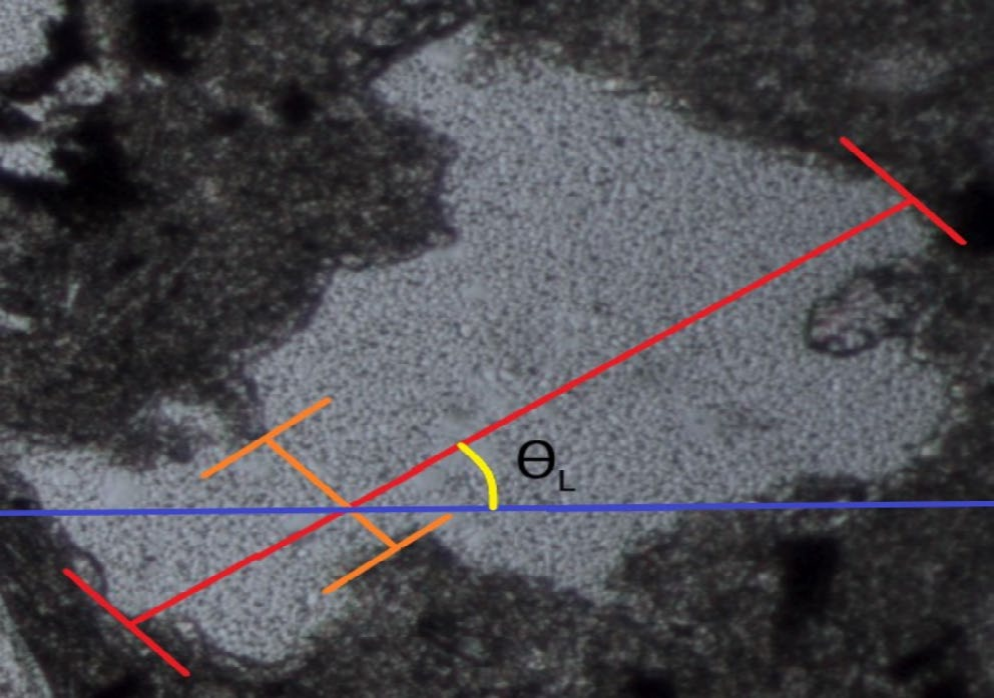
Representation of the angle (𝜃_*L*_) of a grain in any image of a rock.

The value of AF_1_ represents the orientation angle of mineral particles with aspect ratio values greater than 2 placed in the thin section photo. In Figure 4, it is presented how the AF_1_ values are calculated visually. According to Equation (3), each mineral particllongestnded and shortest sides are determined and drawn with suggested software. Then, the angle (𝜃_*L*_) between the longesd the shoaxest axis of mineral particles has been determined with the proposed software.

The angle values are calculated for each texture particle with an aspect ratio greater than 2. Subsequently, combinations of angle differences between these grains have been computed. Each element (*θ*) of the angle difference matrix is provided as an input to the equation given below (Equation 3):

(3) $${\mathrm{AF}}_{1}=\sum_{i=1}^{9}\left(\frac{x_{i}}{\frac{N_{1}\times \left(N_{1}-1\right)}{2}}\right)\times i$$

Thus, $x_{i}$ represents the number of elements in each class, $N_{1}$ is the count of mineral particles with a aspect ratio greater than 2, i is the weight factor and class number.

The relationship between angle differences, weight factors, and class numbers is provided in Table 2. According to this table, weight factors and class numbers are determined.

**TABLE 2:** The relationship between classes and weight in terms of angle differences.

Number	Class boundary	Weight (*i*)
1	θ ≤ 10	1
2	10 < θ ≤ 20	2
3	20 < θ ≤ 30	3
4	30 < θ ≤ 40	4
5	40 < θ ≤ 50	5
6	50 < θ ≤ 60	6
7	60 < θ ≤ 70	7
8	70 < θ ≤ 80	8
9	80 < θ ≤ 90	9

The calculated value of AF_1_ should be divided by five to prevent it from inversely affecting the TC value and to mitigate deviation. This way, the final value of AF_1_ is obtained.

The thin sections of the rock samples used in the study have been examined under a polarized microscope, and their images have been taken and recorded. The acquired thin‐section images were examined with image processing and segmentation algorithms from a novel perspective, leading to the calculation of TC values.

### 2.2 Rock Acquisition and Images Under the Microscope

In this study, 20 rock samples, comprising magmatic, metamorphic, sedimentary rocks, and metallic rocks, were collected from natural stone quarries and natural stone processing factories (Table 3).

**TABLE 3:** Rocks and locations included in the study.

No.	Rock names	Locations
1	Gabbro	Eskişehir/Mihalıççık
2	Granodiorite	Eskişehir/Sivrihisar
3	Hematite	Eskişehir/Sivrihisar
4	Marble‐3	Muğla/Menteşe
5	Biomicrite	Konya/Selçuklu
6	Lead‐Zinc	Niğde/Ulukışla
7	Mikashist	Konya/Yunak
8	Magnesite	Eskişehir/Sivrihisar
9	Marl	Konya/Selçuklu
10	Marble‐1	Konya/Yunak
11	Marble‐2	Konya/Meram
12	Onyx	Eskişehir/Mihalıççık
13	Serpantinite	Eskişehir/Mihalıççık
14	Travertine	Karaman/Taşkale
15	Tuff	Konya/Meram
16	Andesite‐1	Ankara/Gölbaşı
17	Andesite‐2	Konya/Meram
18	Basalt	Kayseri/Kocasinan
19	Dolomite	Konya/Yunak
20	Phonolite	Konya/Seydişehir

**FIGURE 5:**
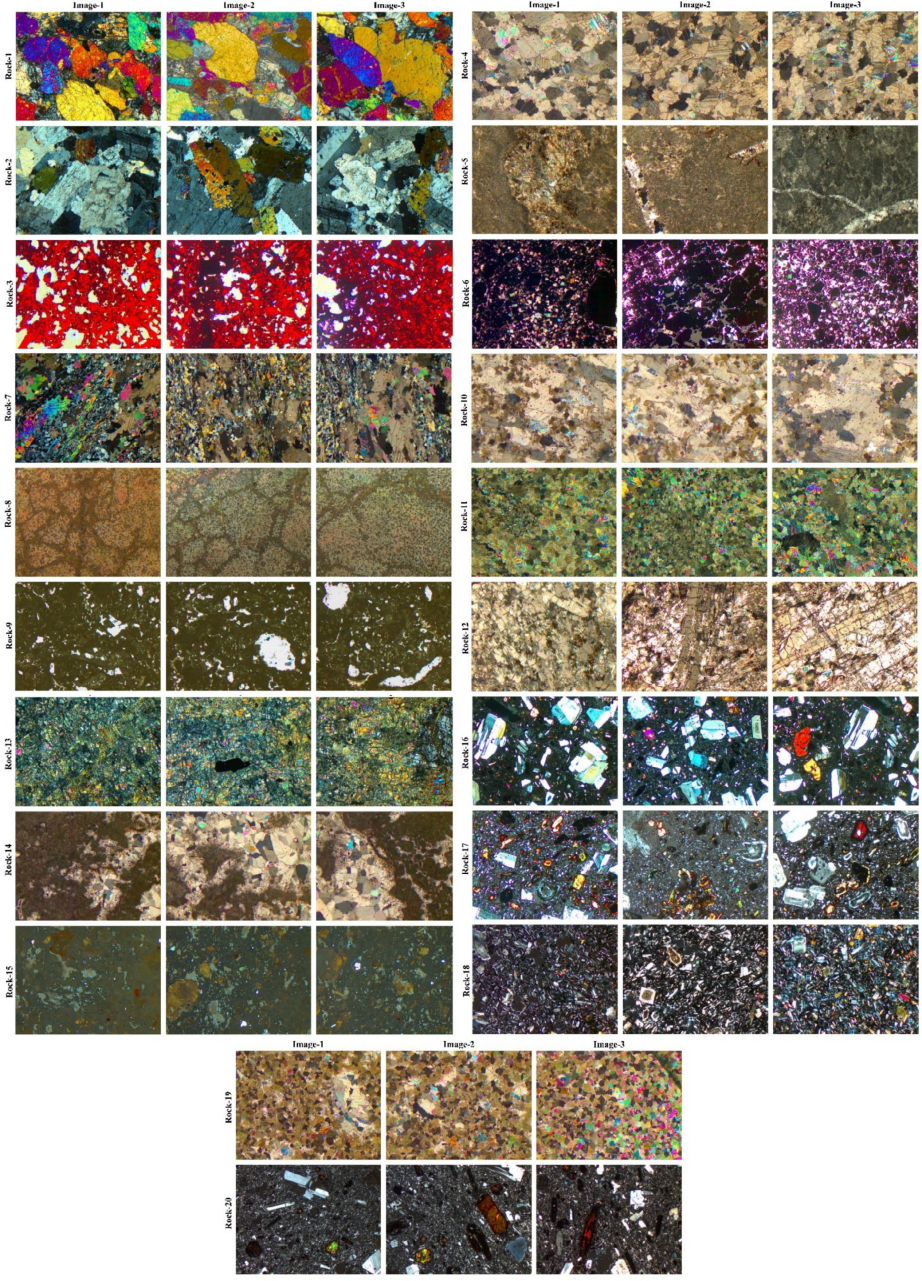
Microscopic images of rock textures (cross‐polarized). (1) Gabbro, (2) Granodiorite, (3) Hematite, (4) Marble‐3, (5) Biomicrite, (6) Lead‐Zinc, (7) Mikashist, (8) Magnesite, (9) Marl, (10) Marble‐1, (11) Marble‐2, (12) Onyx, (13) Serpantinite, (14) Travertine, (15) Tuff, (16) Andesite‐1, (17) Andesite‐2, (18) Basalt, (19) Dolomite, (20) Phonolite.

To characterize the textures of the rock samples used in this study, thin sections were prepared in accordance with the method recommended in TS EN‐12407 (2019) and analyzed under a Leica DM2700 P polarized light microscope. Microscopic images were acquired using a Leica DM2700 P polarizing microscope. Imaging was conducted in transmitted light mode under crossed polarizers, utilizing objective lenses with magnifications of 5×, 10×, 20×, and 40×. A 12 V/30 W halogen lamp served as the light source, and the condenser system was focused using a Bertrand lens. Images were captured with a Leica ICC50 HD CMOS camera (5 MP resolution) and recorded at 1920 × 1080 pixels using Leica Application Suite (LAS) EZ version 3.4.0. White balance and exposure settings were adjusted automatically. Figure 5 includes the names of rock textures collected from 20 different rocks along with the microscopic images.

In the scope of this study, three different texture images have been acquired from each of the thin sections of 20 different rocks, as shown in Figure 5. These texture images have been gathered with the help of a polarized microscope that contains an attached high‐resolution camera. Crossed‐polarized images encompass all colors displayed by minerals except their complete extinction states.

In this study, crossed images of rock textures were utilized based on the visibility of texture particles. This study has confirmed that the segmentation performance depends on the clarity of texture particles but has not been included within the scope of this work.

### 2.3 İmage Segmentation Algorithm

A deep learning‐based image segmentation algorithm is the most suitable technique for examining microscopic images of rocks and providing segmentation to determine the physical properties of textures (area, perimeter, angle, aspect ratio, etc.). The difficulty in identifying sharp transitions in rock images, the inability to distinguish nested mineral particles, the different colors of texture particles, and the potential for more accurate segmentation results have led to the preference for deep learning‐based image segmentation techniques in this study.

The literature presents a diverse range of deep learning‐based image segmentation techniques, including application‐specific models, pre‐trained networks, and universal segmentation frameworks. In this study, various deep learning‐based segmentation algorithms, such as application‐specific models, pre‐trained networks, and universal segmentation frameworks (e.g., Viso.ai, Segment Anything, and DeepLabV3), were assessed for their effectiveness in segmenting the textures of rock samples in microscopic images. After rigorous evaluation of multiple segmentation algorithms, the Segment Anything Model demonstrated the highest performance in accurately identifying mineral particles within texture images. The performance metrics of this model exhibited outstanding accuracy, achieving near‐perfect segmentation results (Kirillov et al. 2023).

**FIGURE 6:**
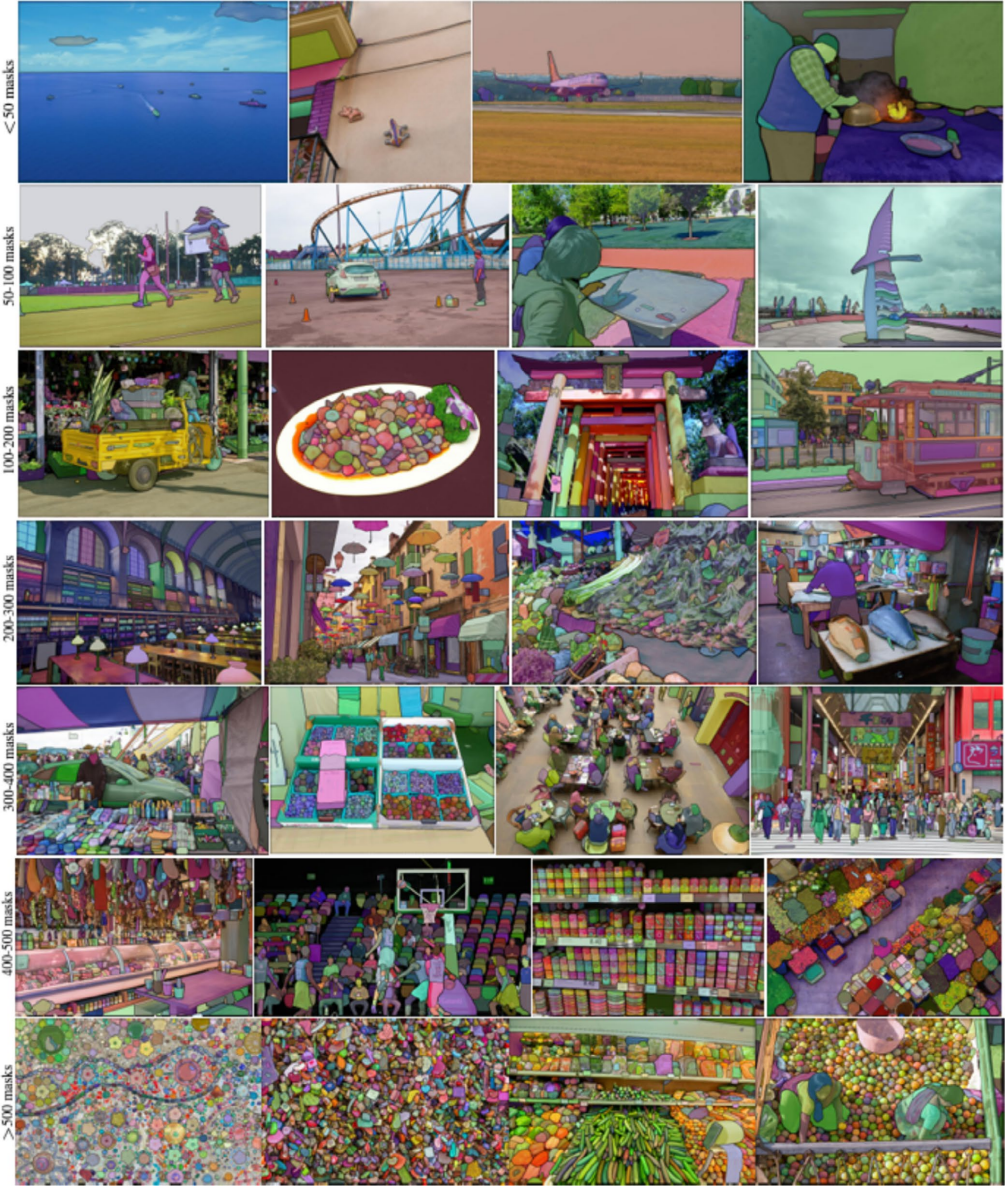
Segmented images obtained using the Segment Anything Model (Kirillov et al. 2023).

The artificial neural network underlying the Segment Anything Model was trained on an extensive dataset comprising approximately 11 million images, with over 1 billion mask generation operations conducted during the training process (Kirillov et al. 2023). In the present study, the model demonstrated exceptional efficacy in analyzing texture granules and extracting relevant features. The segmentation performance metrics, including Intersection over Union (IoU) and stability, have been systematically evaluated and reported. Figure 6 presents representative examples of image segmentation performed using the Segment Anything Model.

As illustrated in Figure 6, the number of masks (i.e., segmented objects) and the masking performance of the Segment Anything Model are clearly demonstrated across different images. One of the key image segmentation evaluation metrics, the mean Intersection over Union (mIoU), has been reported as 95 (Kirillov et al. 2023). This value serves as an indicator of segmentation quality, implying that the Segment Anything Model achieves at least 95% accuracy in segmenting objects within an image.

**FIGURE 7:**
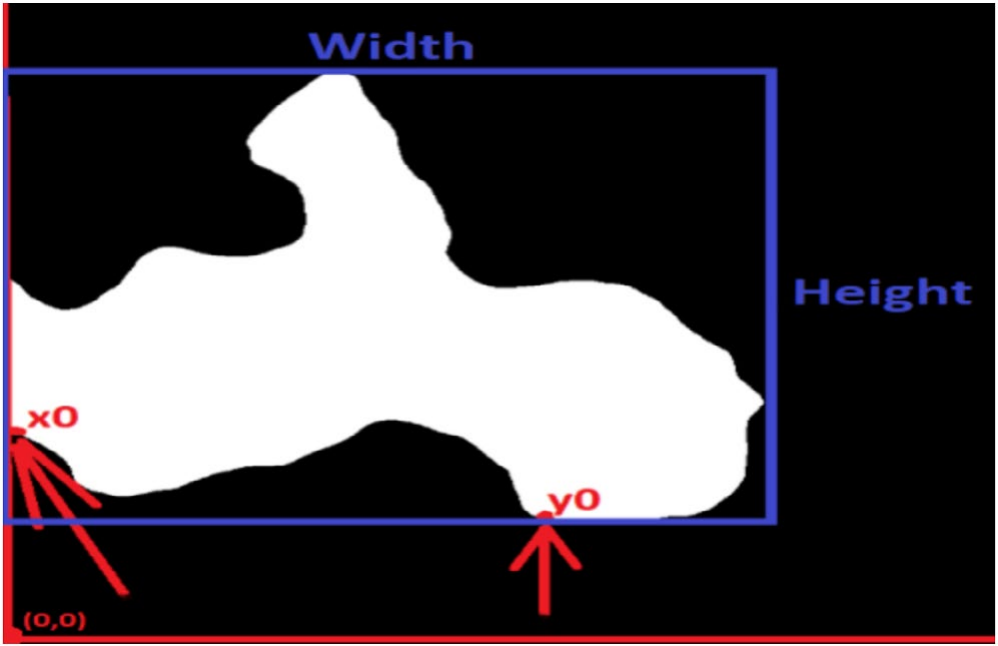
Representation of the *x*0 and *y*0 values on the detected grain after segmentation.

The processes of determining the mineralogical or texture properties of rocks using the Segment Anything Model are summarized below:

1. The rock‐thin section image, which will undergo the segmentation process, is performed with preprocessing techniques, including mean filtering and optimization of contrast and brightness values. These preprocessing techniques have been implemented using the Python programming language with the assistance of the OpenCV library.
2. The goal is to detect textures by using the segment anything deep learning segmentation technique, which has been previously trained with different datasets to create the model. The rock‐thin section image targeted for segmentation is divided into 64 × 32 patches and cropped. Color detection of pixel values is performed for each cropped patch. Based on color detection, the *x*–*y* coordinates of the texture's origin, denoted as *x*0 and *y*0, are determined relative to the origin. The *x*0 point represents the leftmost starting point of the mineral grain in the *x*‐coordinate, while the *y*0 point represents the bottommost starting point in the *y*‐coordinate. Figure 7 visually represents the *x*0 and *y*0 values indicating the detected mineral grain properties in the rock thin section image.

The *x*0 and *y*0 values are determined. The highest and the widest points of the rock texture image are identified to determine the width and height of the grain. Figure 2 represents the width and height of the detected mineral grains. The width and height values are divided to calculate the aspect ratio.

3 After calculating the aspect ratio of the detected mineral grains using the OpenCV library, the image's area, perimeter, and mineral grain orientation are determined through contours. The Python OpenCV library plays a crucial role in determining these values, and the written code helps store and save the physical characteristics of each mineral grain's properties in the memory.

**FIGURE 8:**
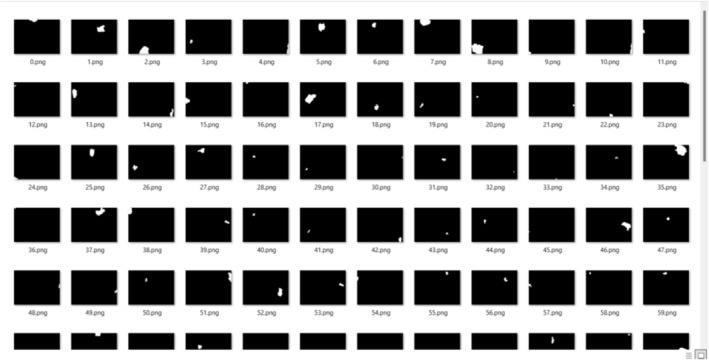
Representation of segmented texture particles for a known rock.

Using the Segment Anything Model, known rock in the study, provided here as an example to explain the subject, has undergone the segmentation process. The original texture of this rock contains 137 particles, and the segmentation of these 137 particles has been achieved with an accuracy of 97.7% ± 0.010%. Figure 8 shows some texture images obtained from the segmentation results.

### 2.4 Calculation of TC With Image Processing Algorithm

**FIGURE 9:**
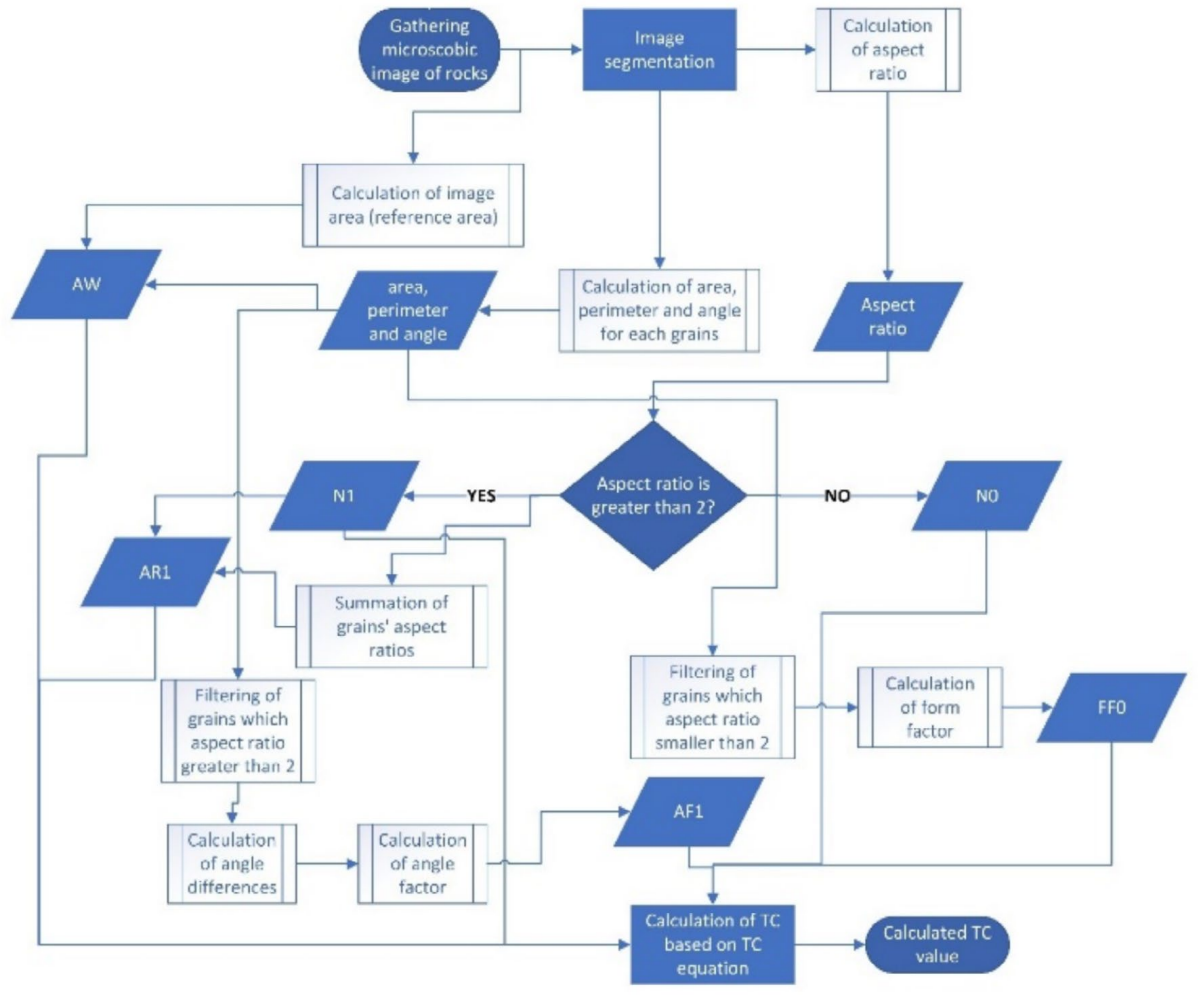
Algorithm flow diagram for calculating TC.

The TC values of 20 rock samples have been calculated using the proposed method. This calculation process was implemented using Python software. The algorithm flow diagram for calculating the TC has been presented in Figure 9.

**FIGURE 10:**
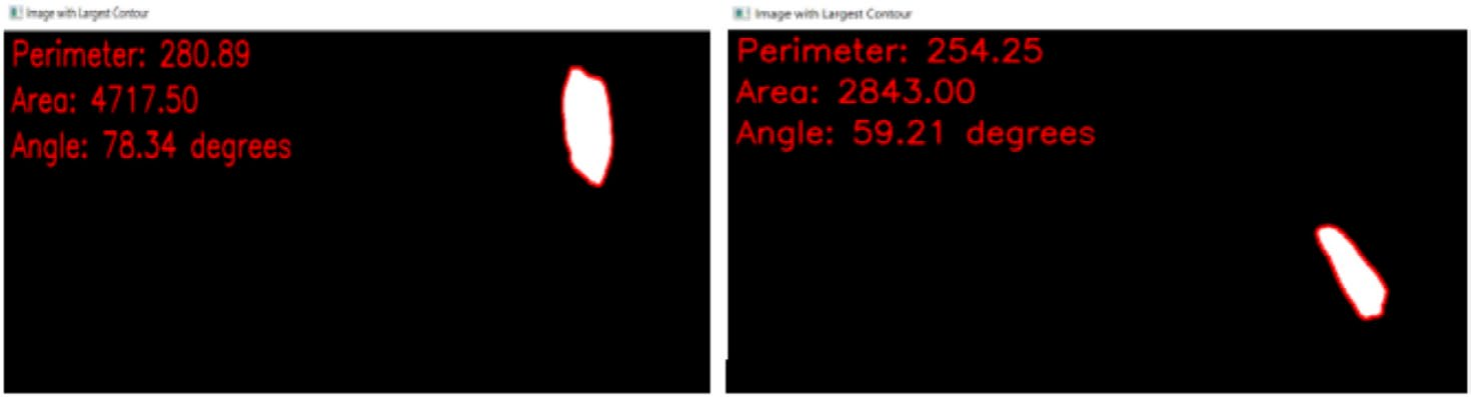
The representation of perimeter, area, and angle values for the segmented grain in texture image using the developed software.

The first step in the flowchart given above for calculating the TC value is acquiring microscopic images of rock textures. Subsequently, the grains were segmented using the segmentation technique provided in Section 2.4. In the example of a known rock given in Figure 7, 137 segmented grains were detected. The aspect ratio of each of these grains was calculated and recorded. The numerical value of the aspect ratio was used to determine the number of particles, *N*_1_, with a numerical value greater than 2, and the number of particles, *N*_0_, with a numerical value less than 2. The physical properties of the detected grains, such as area, angle, and perimeter, were calculated using the code developed with the algorithm provided in Figures 8 and 10 illustrates the calculation of the perimeter, area, and angle information for 2 grains.

After calculating the area of each mineral particle in the segmented grain image, the area of each grain was summed and saved. The area of the segmented image (reference area) was calculated using the developed code by dividing the total area of the detected grains.

The AW values have been calculated by dividing the total area of the detected grains by the reference area. The value was calculated and saved by summing the aspect ratios of grains with a numerical aspect ratio greater than 2 and dividing them by *N*_1_.

The angle factor (AF_1_) value was calculated using the formula given in Equation (3) after determining the angle difference combinations of grains with a numerical aspect ratio greater than 2. The form factor (FF_0_) value was calculated using the formula given in Equation (2) for each grain with a numerical aspect ratio less than 2 and whose area and perimeter were determined.

**FIGURE 11:**
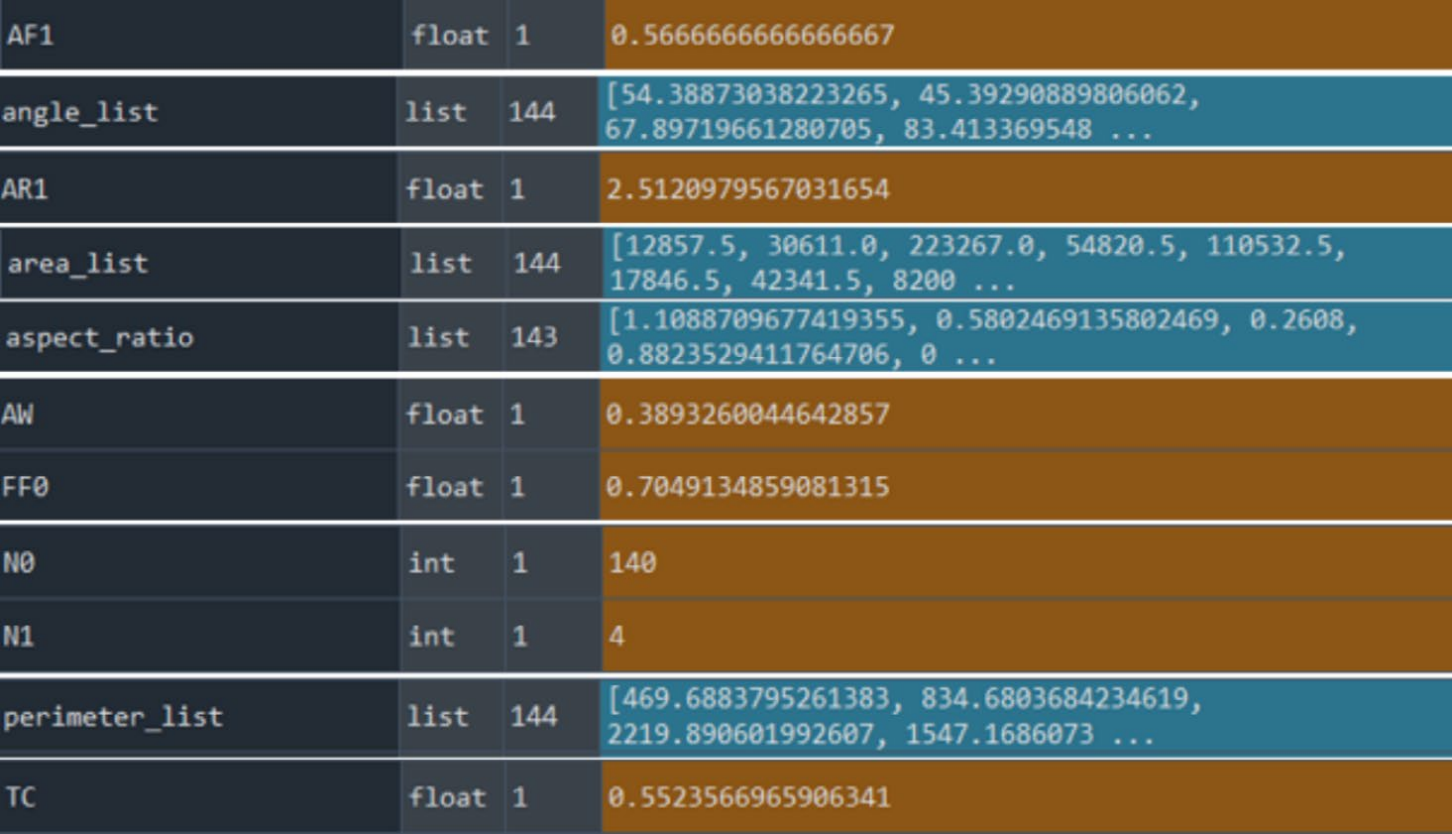
The representation of parameters calculated and recorded with the code developed for the calculation of TC value of a known rock's microscopic image.

Finally, the TC value was calculated using the parameters AW, *N*_1_, *N*_0_, AF_1_, FF_0_ and AR_1_ obtained by substituting them into the formula in Equation (1). Figure 11 shows the parameters required for calculating the TC value for the example of a rock image obtained using the written code.

### 2.5 Automated Calculation of TC in Rock Samples Using Image Segmentation and Processing Techniques: Results

**FIGURE 12:**
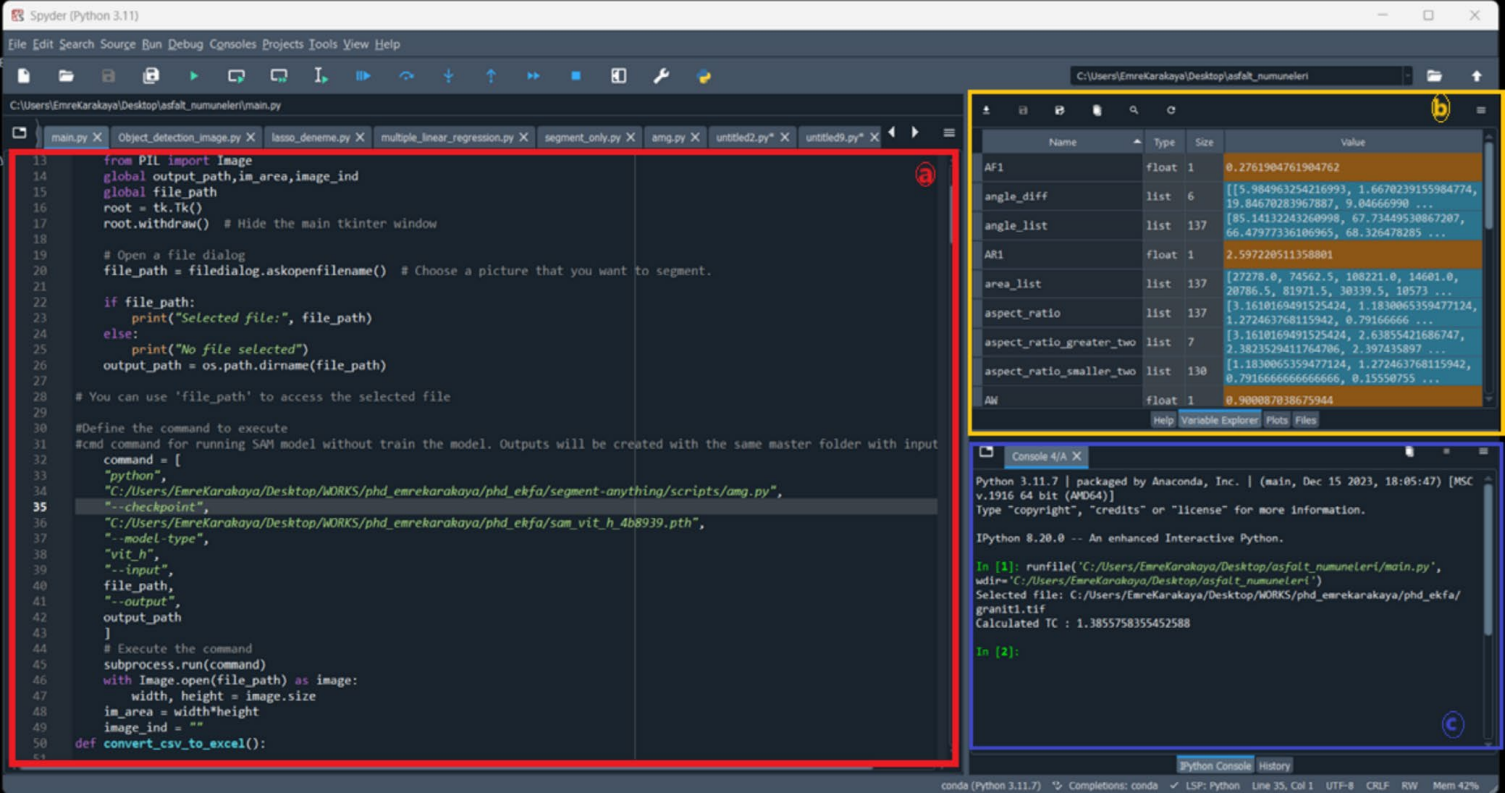
The code developed for calculating the texture coefficient (a), the computed texture coefficient representation (b), and the visualization of each parameter in the texture coefficient equation (c) are presented.

The TC values of the 20 rock texture images included in the study were calculated using the code developed considering the image segmentation and image processing information described in Sections 2.4 and 2.5. Figure 12 illustrates the Python code developed for computing the TC of rock samples (a) and the TC calculation for the Granodiorite‐1 rock sample analyzed in this study (c). Additionally, the program includes a dedicated section that provides the individual results for each parameter in the TC equation (AW, *N*_0_, *N*_1_, AF_1_, AR_1_, FF_0_), ensuring a comprehensive breakdown of the computed values (c).

**FIGURE 13:**
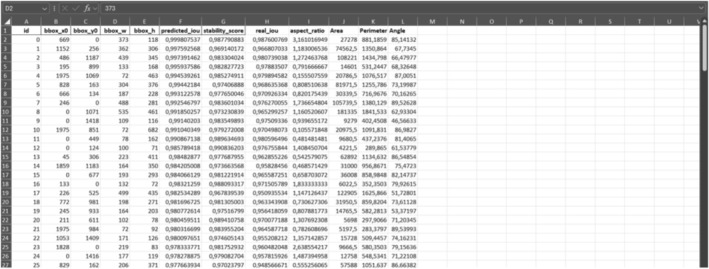
The physical properties of each mineral identified in the texture image of the Granodiorite‐1 sample are presented, including the following parameters: Id (the identification number of segmented minerals), bbox_x0 (the *x*‐coordinate of the leftmost starting point in the *x*–*y* coordinate system), bbox_y0 (the *y*‐coordinate of the bottommost starting point in the *x*–*y* coordinate system), bbox_w (the width of the mineral), bbox_h (the height of the mineral), predicted_iou (the predicted intersection‐over‐union value representing the overlap between the detected and ground‐truth bounding boxes), stability_score (the stability score of the detected mineral), real_iou (the actual intersection‐over‐union value of the detected bounding boxes), aspect_ratio (the width‐to‐height ratio of the mineral), area (the total area of the mineral), perimeter (the perimeter of the mineral), and angle (the orientation angle of the mineral).

The developed program additionally generates an Excel file that provides detailed physical attributes of each mineral within the analyzed rock sample, including parameters such as width, length, area, angle, and perimeter. Figure 13 illustrates the physical characteristics of individual minerals present in the texture image of the Granodiorite‐1 sample.

The calculated TC values and other parameters mentioned in Equation (1) are presented in Table 4. In this table, three different grain images are gathered from each thin‐section rock image, and the TC and other parameters in Equation (1) were calculated for each image.

**TABLE 4:** The TC parameters and calculated TC values for the rock texture images included in the study.

Rock name	AW	*N*_0_	*N*_1_	AR_1_	AF_1_	FF_0_	TC	Average TC
Gabbro‐1	0.91	75	5	2.94	0.2	0.62	1.4	1.29 ± 0.1
Gabbro‐2	0.9	91	14	3.6	0.25	0.64	1.33	
Gabbro‐3	0.8	104	10	2.92	0.2	0.66	1.14	
Granodiorite‐1	0.9	130	7	2.59	0.27	0.63	1.38	1.32 ± 0.04
Granodiorite‐2	0.88	131	4	2.3	0.4	0.65	1.32	
Granodiorite‐3	0.84	150	6	2.64	0.22	0.64	1.28	
Tuff‐1	0.21	175	6	2.51	0.27	0.66	0.31	0.38 ± 0.05
Tuff∐2	0.29	186	2	2.16	0.2	0.68	0.42	
Tuff∐3	0.3	224	7	2.38	0.23	0.69	0.42	
Biomicrite‐1	0.31	21	1	2.08	0	0.71	0.42	0.39 ± 0.03
Biomicrite‐2	0.31	42	2	2.44	0.2	0.73	0.41	
Biomicrite‐3	0.25	60	3	2.66	0.2	0.69	0.34	
Marl‐1	0.12	110	6	2.67	0.24	0.64	0.18	0.24 ± 0.04
Marl‐2	0.17	107	6	2.86	0.3	0.64	0.26	
Marl‐3	0.19	118	3	2.61	0.4	0.68	0.28	
Andesite‐1	0.48	206	4	2.26	0.26	0.68	0.69	0.69 ± 0.04
Andesite‐2	0.48	240	7	2.38	0.41	0.74	0.64	
Andesite‐3	0.54	232	2	2.3	0.2	0.71	0.76	
Andesite2‐1	0.39	233	14	2.89	0.23	0.73	0.51	0.53 ± 0.13
Andesite2‐2	0.29	234	20	2.43	0.26	0.72	0.38	
Andesite2‐3	0.52	245	14	2.54	0.22	0.71	0.7	
Marble‐1	0.49	197	10	2.91	0.22	0.72	0.67	0.8 ± 0.09
Marble‐2	0.63	256	13	3.05	0.23	0.69	0.88	
Marble‐3	0.63	185	10	2.47	0.21	0.7	0.86	
Marble2‐1	0.61	253	11	2.57	0.2	0.7	0.84	0.74 ± 0.15
Marble2‐2	0.38	266	1	2.11	0	0.72	0.52	
Marble2‐3	0.59	220	6	2.35	0.2	0.68	0.86	
Marble3‐1	0.86	271	12	2.81	0.29	0.65	1.28	1.19 ± 0.05
Marble3‐2	0.8	256	18	2.5	0.24	0.66	1.15	
Marble3‐3	0.8	300	14	2.55	0.27	0.67	1.16	
Serpantinite‐1	0.14	193	1	2.4	0	0.75	0.19	0.22 ± 0.02
Serpantinite‐1	0.17	188	3	2.47	0.2	0.77	0.22	
Serpantinite‐1	0.2	157	4	2.58	0.2	0.78	0.25	
Magnesite‐1	There are no grains							0.44
Magnesite‐2	0.35	37	2	2.48	0.2	0.76	0.44	
Magnesite‐3	There are no grains							
Basalt‐1	0.28	318	22	2.55	0.22	0.67	0.4	0.39 ± 0.02
Basalt‐2	0.28	284	13	2.45	0.2	0.66	0.41	
Basalt‐3	0.25	315	14	2.8	0.21	0.67	0.36	
Lead‐Zinc‐1	0.3	176	3	2.34	0.2	0.71	0.42	0.51 ± 0.08
Lead‐Zinc‐2	0.39	153	9	2.64	0.31	0.6	0.62	
Lead‐Zinc‐3	0.35	292	4	2.19	0.23	0.67	0.51	
Dolomite‐1	0.65	370	5	2.51	0.2	0.69	0.93	0.98 ± 0.04
Dolomite‐2	0.72	379	9	2.37	0.2	0.69	1.02	
Dolomite‐3	0.72	477	6	2.57	0.2	0.7	1.01	
Travertine‐1	0.4	105	6	2.44	0.2	0.67	0.58	1.04 ± 0.3
Travertine‐2	0.85	264	7	2.38	0.2	0.68	1.22	
Travertine‐3	0.95	131	7	2.85	0.21	0.7	1.32	
Onyx‐1	0.42	222	3	2.57	0.26	0.68	0.61	0.61 ± 0
Onyx‐2	0.42	179	7	2.54	0.23	0.68	0.61	
Onyx‐3	0.45	214	10	2.52	0.23	0.71	0.61	
Phonolite‐1	0.33	170	10	2.73	0.26	0.71	0.45	0.48 ± 0.04
Phonolite‐2	0.38	163	5	2.91	0.25	0.7	0.54	
Phonolite‐3	0.33	172	5	2.49	0.2	0.71	0.46	
Mikashist‐1	0.49	299	7	2.31	0.2	0.68	0.62	0.69 ± 0.06
Mikashist‐1	0.47	269	1	2.33	0	0.68	0.68	
Mikashist‐1	0.52	287	1	2.29	0	0.66	0.78	
Hematite‐1	0.43	195	6	2.69	0.2	0.59	0.71	0.53 ± 0.12
Hematite‐2	0.3	231	8	2.46	0.37	0.63	0.47	
Hematite‐3	0.27	277	1	2.09	0	0.67	0.41	

Table 5 presents the TC values of rock types found in the literature that are similar to those examined in this study, along with their locations and the software used for TC computation. As observed in Table 5, the TC values reported for the same rock types exhibit variations. Even for rocks of identical type and location (Uşak/Karahallı‐Marble), discrepancies in TC values are evident. Consequently, the TC values computed using the proposed method should not be expected to be identical or entirely consistent with those reported for similar rocks in Table 5. Nevertheless, a general similarity is observed between the TC values of the rocks analyzed in this study and those reported in the literature for comparable rock types.

**TABLE 5:** TC values of some types of rocks, their locations, and software/methodology in the literature.

References	Software/methodology	Rock type	Location	TC
Ozturk and Nasuf (2013)	Fuzzy Logic	Travertine	Konya/Karaman	0.85
		Travertine	İcel/Mut	0.9
		Travertine	Antalya/Demre	0.56
		Travertine	Antalya/Finike	1.01
		Travertine	Burdur/Bucak	0.62
		Travertine	Antalya/Demre	1.03
		Serpentine	Konya	0.4
		Marl	Gaziantep/Erikli	0.46
		Marl	Kütahya/Tunçbilek	0.16
		Marble	Antalya/Korkuteli	1.22
Esmailzadeh et al. (2017)	AutoCAD	Red Travertine	Unknown	0.94
		Khalkhal Travertine	Unknown	0.91
Tumac et al. (2018)	AutoCAD	Marble	Muğla/Yatağan	3.1
		Marble	Muğla/Yatağan	2.8
		Marble	Muğla/Ula	3.3
		Marble	Muğla/Ula	2.9
		Marble	Bursa/Mustafa Kemal Paşa	2.7
		Marble	Uşak/Karahallı	1.1
		Marble	Uşak/Karahallı	1.8
		Marble	Uşak/Karahallı	2.3
		Marble	Uşak/Sivaslı	1.8
		Marble	Muğla/Yatağan	2.7
Comakli and Cayirli (2019)	Image Pro Plus	Tracy Andesite	Niğde	3.26
		Basaltic Andesite	Ankara	2.11
		Marble	Aksaray	2.45
		Marble	Afyon	2.53
		Marble	Denizli	2.61
		Marble	Niğde	2.18
Atici and Comakli (2019)	Image Pro Plus	Diorite	Samsun	1.25

### 2.6 The Importance of the AW Parameter in the TC Equation

The AW parameter reflects structural variations arising from the distinct weight distributions of minerals within the rock, thereby enhancing the TC's sensitivity to the rock's actual physical and mechanical properties. Furthermore, AW contributes to the more precise classification of rock textures in data obtained through image processing and segmentation techniques, consequently improving the reliability of the calculated TC values. Additionally, the incorporation of AW enables a more effective assessment of textural differences among igneous, sedimentary, and metamorphic rocks. As a result, the AW parameter plays a crucial role in the more comprehensive and reliable calculation of the TC, thereby facilitating a more accurate prediction of the engineering properties of rocks.

**FIGURE 14:**
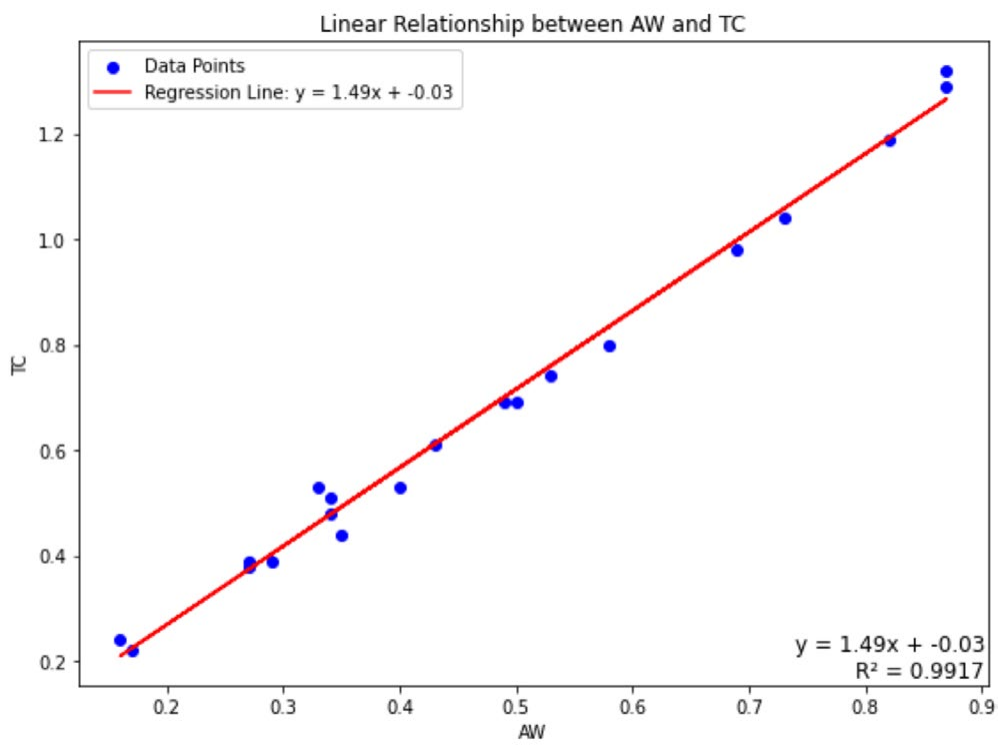
The relationship between TC and the average of AW values of each grain in its own group.

The AW parameter in the TC equation is derived by dividing the total area of rock particles in the image by the total area of the entire image. Certain rocks exhibit lower AW values than expected, resulting in correspondingly lower TC values. The relationship between the TC and AW values of the rocks in this study is presented in Figure 14. The AW values for each individual grain image were calculated relative to the average of their respective group. A lower AW value indicates that the matrix (the region devoid of rock particles) occupies a larger area. In some rocks, such as basalt, a low crystallization rate leads to a reduced AW value. Consequently, a lower AW value corresponds to a lower TC value.

According to Figure 14, the relationship between the AW and TC values is exceptionally high (*R* ^2^: 0.9917). In summary, it has been determined that calculating the AW value of the thin‐section image of a given rock is sufficient to compute its corresponding TC value.

## 3 Conclusions and Discussions

Rock mechanics studies are crucial, especially in the mining sector, geology, and construction. TC value provides important information about a rock's strength and drilling rate, making it a significant parameter. Therefore, calculating the TC value accurately is very important for rock mechanics studies. In this study, microscopic images obtained from thin sections of 20 rock samples were subjected to segmentation using a deep learning‐based image processing technique, and a novel Python‐based algorithm was developed for TC calculations. The results obtained are summarized below.

- Accurate determination of a rock's TC is critically dependent on the flawless segmentation of each particle within its texture. Identifying every particle in a rock texture image and correctly segmenting them to yield precise segmentation outputs is directly linked to the accurate calculation of the parameters in the TC equation.
- This study conducted a comprehensive evaluation of various deep learning‐based segmentation algorithms, including application‐specific models, pre‐trained networks, and universal segmentation frameworks (e.g., Viso.ai, Segment Anything, and DeepLabV3), to assess their efficacy in segmenting the textures of rock samples in microscopic imagery. Following an extensive comparative analysis, the Segment Anything Model exhibited the highest accuracy in delineating mineral particles within texture images, demonstrating its superior capability in precise and reliable segmentation.
- The TC equation encompasses the physical properties (such as width, length, area, perimeter, and angle) of each rock particle within the texture image. By accurately calculating the physical characteristics of every rock particle in the texture image, the final TC value is derived.
- High segmentation performance reflects the precision of TC calculations. The method proposed for calculating TC values in rocks is highly original and yields exceptionally high segmentation accuracy (IoU = 0.97). Using this method, the calculation of TC for any rock takes less than a minute. Furthermore, this method can be applied not only to calculate the TC of rocks but also, if microscopic images are provided of any type of material, it can easily calculate and analyze the TCs and physical properties of any material.
- The relationship between the AW parameter and TC values is extraordinarily high (*R* ^2^: 0.9917), indicating that the TC value of any rock is almost equal to its AW value.
- The TC values of rock samples collected from the same type and location may exhibit variations. The primary reasons for this discrepancy include differences in the mineralogical and petrographic composition of the rocks, variations in the exact sampling location, differences in the methodology or software used for TC calculation, variations in the microscopic image quality of the rock textures, and whether segmentation is performed manually or automatically. Additionally, in microscopic images, the manual delineation of rock texture boundaries introduces a human factor, further contributing to discrepancies. Furthermore, the anisotropic nature of the rock samples can also influence TC values.

In rocks with clear and defined texture boundaries, the segmentation process is more functional, allowing for a more detailed differentiation. However, in rocks where the texture boundaries are unclear (such as magnesite) or those containing a high proportion of volcanic glass (e.g., some basaltic rocks), the matrix yields a very low value. The matrix is directly related to the AW parameter. For rocks with high strength but low AW values, it is recommended that a new AW coefficient be determined.

This study employs an automated method for calculating the TC values of rocks, aiming to minimize human intervention and achieve more accurate results. Previous studies in the literature have indicated that the delineation of texture boundaries often involves significant human interaction, leading to variations in TC values depending on the researcher's expertise. This, in turn, increases susceptibility to errors and inconsistencies in TC computations. The proposed method/software seeks to address this issue by providing a standardized and objective approach.

In practical applications, the proposed method can be employed for precise and reliable TC calculations due to its minimal human involvement, ease of use, and ability to produce rapid results. Regardless of the specific methodology applied, variations in TC values have been observed even among rock samples of the same type and location. Therefore, to enhance the accuracy of the proposed approach, many thin‐section samples should be collected from the same rock and location, and their TC values should be subjected to statistical analysis.

Given the rapid advancements in technology and artificial intelligence, future research can build upon this study by integrating more sophisticated image processing and segmentation techniques. As a result, more precise texture boundary delineations could be achieved, ultimately improving the accuracy of AW and TC calculations and further enhancing the proposed method.

## Author Contributions

**Emre Karakaya:** conceptualization, methodology, software, supervision, resources, project administration, investigation, writing – original draft, writing – review and editing, visualization, validation, formal analysis, data curation, funding acquisition. **Bilgehan Kekeç:** conceptualization, methodology, supervision, project administration. **Niyazi Bilim:** conceptualization, methodology, project administration, supervision. **Fatih V. Adigözel:** conceptualization, methodology, software, investigation, validation, formal analysis, data curation, supervision, visualization, project administration, resources, writing – original draft, writing – review and editing.

## Conflicts of Interest

The authors declare no conflicts of interest.

## Data Availability

The data that support the findings of this study are available from the corresponding author upon reasonable request.
